# The association between microvascular and macrovascular endothelial function in patients with rheumatoid arthritis: a cross-sectional study

**DOI:** 10.1186/ar3374

**Published:** 2011-06-21

**Authors:** Aamer Sandoo, Douglas Carroll, George S Metsios, George D Kitas, Jet JCS Veldhuijzen van Zanten

**Affiliations:** 1 Department of Rheumatology, Dudley Group of Hospitals NHS Trust, Russells Hall Hospital, Pensnett Road, Dudley, DY1 2HQ, West Midlands, UK; 2 School of Sport and Exercise Sciences, University of Birmingham, Edgbaston, Birmingham, B15 2TT, UK; 3 Arthritis Research UK Epidemiology Unit, University of Manchester, Oxford Road, Manchester, M13 9PL, UK

## Abstract

**Introduction:**

Patients with rheumatoid arthritis (RA) are at an increased risk for cardiovascular disease (CVD). One of the earliest manifestations of CVD is endothelial dysfunction (ED). ED can occur in both the microcirculation and the macrocirculation, and these manifestations might be relatively independent of each other. Little is known about the association between endothelial function in the microcirculation and the macrocirculation in RA. The objectives of the present study were to examine the relationship between microvascular and macrovascular endothelial function in patients with RA.

**Methods:**

Ninety-nine RA patients (72 females, mean age (± SD) 56 ± 12 years), underwent assessments of endothelial-dependent (acetylcholine) and endothelial-independent (sodium nitroprusside) microvascular vasodilatory function (laser Doppler imaging with iontophoresis), as well as endothelial-dependent (flow-mediated dilation) and endothelial-independent (glyceryl trinitrate-mediated dilation) macrovascular vasodilatory function. Vasodilatory function was calculated as the percentage increase after each stimulus was applied relative to baseline values.

**Results:**

Pearson correlations showed that microvascular endothelial-dependent function was not associated with macrovascular endothelial-dependent function (*r *(90 patients) = 0.10, *P *= 0.34). Similarly, microvascular endothelial-independent function was not related to macrovascular endothelial-independent function (*r *(89 patients) = 0.00, *P *= 0.99).

**Conclusions:**

Microvascular and macrovascular endothelial function were independent of each other in patients with RA, suggesting differential regulation of endothelial function in these two vascular beds. Assessments of both vascular beds may provide more meaningful clinical information on vascular risk in RA, but this hypothesis needs to be confirmed in long-term prospective studies.

## Introduction

The endothelium is the innermost lining of the vasculature and is responsible for maintaining vascular homeostasis via the balanced production of a number of vasoactive factors [1]. One such factor is nitric oxide (NO), which plays an important role in vasodilation and in inhibiting atherosclerotic processes such as thrombosis and leukocyte activation in the vessel wall [2-5]. Damage to the endothelium can reduce NO activity, causing endothelial dysfunction (ED), which is an early indicator of cardiovascular disease (CVD) [6]. The extent of ED can be characterised by assessing endothelial function in different vascular beds of the peripheral circulation [7]. These assessments are reflective of coronary endothelial function [8-11] and have been shown to be good predictors of long-term cardiovascular events in individuals with atherosclerosis [12-15] and peripheral vascular disease [16], as well as in healthy older participants [17].

Endothelial cells can differ in structure and phenotype, depending on the vessel type [18]. Heterogeneous responses to *in vitro *stimulation are displayed in different vascular beds and even in different sections of the same vascular bed [19-21]. This suggests that ED may occur differentially in different vascular beds [21]. Studies that have assessed associations between peripheral microvascular and macrovascular endothelial-dependent function in healthy individuals have reported mixed findings, with some reporting an association between microvascular and macrovascular endothelial-dependent function [22,23] and others reporting no association [24-26].

Rheumatoid arthritis (RA) is a chronic systemic inflammatory disease of the joints [27]. RA patients are also at an increased risk for CVD [28], with ED believed to be a contributor to some of this excess CVD risk [29]. RA has a similar CVD risk burden and vascular profile to diabetes [30], a condition in which microvascular disease may contribute to macrovascular disease [31]. There is also some preliminary evidence that coronary microvascular disease may be apparent before macrovascular disease in RA [32]. This highlights the importance of assessing endothelial function in multiple vascular beds.

To our knowledge, only two studies have simultaneously assessed microvascular and macrovascular endothelial function in RA; one study reported no association between the two vascular beds [33], while the other study did find an association [34]. In the former study, microvascular endothelial function was measured using laser Doppler flowmetry, which assesses endothelial function at a single point only and does not account for spatial heterogeneity of skin blood flow in the way that newer techniques such as laser Doppler imaging (LDI) do [35]. A limitation of the second study is that manual methods were used to detect and mark out the vessel diameter during flow-mediated dilation (FMD). This method is less accurate than automated wall tracking software, which detects and calculates arterial diameter in real time and greatly reduces the variability found in the measurements [36,37]. Such limitations suggest that the association between microvascular and macrovascular endothelial function requires further investigation using newer and more accurate assessments of endothelial function. Accordingly, the aim of the present study was to examine the relationship between microvascular and macrovascular endothelium-dependent function in RA using LDI and automated measurements of vascular diameter changes to reactive hyperaemia.

## Material and methods

### Patients

Ninety-nine consecutive rheumatoid arthritis (RA) patients were recruited from the rheumatology outpatient clinics of the Dudley Group of Hospitals NHS Trust, UK. All patients met the retrospective application of the 1987 revised RA criteria of the American Rheumatism Association [38]. Patients were excluded if they had previously confirmed acute coronary syndrome or established CVD as indicated in their medical records and/or upon questioning during the initial consultation. The study received local Research Ethics Committee approval, and all participants gave their written informed consent according to the Declaration of Helsinki.

### Protocol

Patients reported to a temperature-controlled vascular laboratory (22°C) after a 12-hour overnight fast. For ethical reasons, patients were not asked to refrain from taking RA disease-related or vasoactive medications. All patients underwent a detailed clinical examination, and demographic information was collected from all the participants by questionnaire. The disease activity score in 28 joints [39] was also calculated. Following this step, the participants underwent assessments of microvascular endothelial function using LDI with iontophoresis and assessment of macrovascular endothelial function using FMD and glyceryl trinitrate-mediated dilation (GTN).

### Microvascular endothelial function

Endothelial function of the microvasculature was assessed noninvasively using LDI (moorLDI2 SIM; Moor Instruments Ltd, Devon, UK) with iontophoresis of 1% acetylcholine (Ach; endothelium-dependent) and 1% sodium nitroprusside (SNP; endothelium-independent) (Sigma Chemical Co., Montvale, NJ, USA) in 0.5 mL of saline by a single observer (AS). The technique was performed according to previously established guidelines [35] and was described in detail previously [40]. Briefly, after a baseline scan, ten scans were recorded during iontophoresis of the vasoactive agents using a 30 μA current, followed by two scans during recovery. This technique has intraobserver coefficients of variation (CVs) of 6.5% and 5.9% for ACh and SNP, respectively, in our laboratory.

### Macrovascular endothelial function

Assessment of macrovascular endothelial-dependent function was performed using FMD with high-resolution ultrasonography of the brachial artery (ACUSON Antares ultrasound system; Siemens PLC, Camberley, UK) according to previously established guidelines [41]. Following ten minutes of rest, endothelium-independent responses were examined by administration of a 500-μg sublingual glyceryl trinitrate tablet (Alpharma, Barnstaple, UK) while the brachial artery was imaged continuously for five minutes. The intraobserver CVs were 10.7% for FMD and 11.8% for GTN assessments, respectively. For all vascular tests, endothelial function was expressed as the percentage increase in perfusion or diameter from baseline, and all analysis was carried out offline by AS, who was blinded to the identity of the patient.

### Statistical analysis

Statistical analysis was performed using SPSS version 16 software (SPSS Inc, Chicago, IL, USA). Variables were tested for normality by using the Kolmogorov-Smirnov test. Log transformation was performed for positively skewed variables as appropriate. Values are expressed as medians (25th to 75th percentiles) or percentages as appropriate. Pearson's correlations were used to assess the relationships between microvascular and macrovascular endothelium-dependent function.

## Results

The patient characteristics are presented in Table 1. The majority of patients were female and had moderate disease activity levels. The percentage increase in blood flow in response to ACh was 311 ± 234, and for SNP it was 306 ± 199. For macrovascular endothelial function, the percentage increase in diameter (FMD) after reactive hyperaemia was 9 ± 6, and the percentage increase in diameter after GTN was 23 ± 9. Microvascular and macrovascular endothelial function for RA patients were similar to a healthy control group (data not reported).

**Table 1:** General and disease-related characteristics for the RA patients^a^

Characteristics	RA patient data
General characteristics	
Age, years	57 (47 to 65)
Females, %	73%
Body mass index	29 (25 to 34)
Disease-related characteristics	
Disease duration, years	8 (3 to 16)
Rheumatoid factor-positive, %	78%
DAS28 score	3.6 (2.5 to 4.6)
C-reactive protein level, mg/L	5 (2.9 to 13.5)
Erythrocyte sedimentation rate, mm/hour)	17 (8.8 to 28.3)

^a^DAS28 = disease activity score in 28 joints; RA = rheumatoid arthritis. Results are expressed as medians (25th to 75th percentiles).

As shown in Table 2, microvascular endothelium-dependent function was not associated with macrovascular endothelium-dependent function. This was also the case with regard to associations between endothelium-independent function in these two vascular beds.

**Table 2:** Correlations between the microvasculature and macrovasculature^a^

Measurement	Endothelial-dependent (FMD)	Endothelial-independent (GTN)
Endothelial-dependent (ACh)	*r *(90) = 0.10, *P *= 0.34	-
Endothelial-independent (SNP)	-	*r *(89) = -0.00, *P *= 0.99

^a^ACh = acetylcholine; SNP = sodium nitroprusside; FMD = flow-mediated dilation; GTN = glyceryl trinitrate-mediated dilation.

## Discussion

In the present study, we found that in RA patients, microvascular and macrovascular endothelial function are not associated with each other. To our knowledge, only two other studies have examined associations between small-and large-vessel endothelial function in RA patients. One study of 65 RA patients used laser Doppler flowmetry to assess microvascular blood flow and reported findings similar to those of the current study [33], whereas another study of 66 RA patients reported that microvascular and macrovascular endothelium-dependent function were only moderately associated with each other [34].

Arosio and colleagues [33] examined both microvascular and macrovascular endothelial function by eliciting reactive hyperaemia. Even though both assessments were dependent on shear stress, microvascular and macrovascular endothelial function were not associated with each other [33], which could be due to a difference in exposure to shear stress between the resistance and conduit vessels [42]. Given that the magnitude of shear stress is directly linked to NO release [43], it is possible that differences in shear stress profiles resulted in the lack of association between these two assessments in the study by Arosio and colleagues [33]. Therefore, it is possible that microvascular and macrovascular endothelial function may differ even when a similar stimulus is used.

Foster and colleagues [34] used the same assessments as we used in the present study [34], but the iontophoresis protocol used to administer the vasoactive agents differed between studies in terms of relative administration of the iontophoresis agents (simultaneously in the current study versus consecutively in the previous study), as did the iontophoresis current delivery (30 μA vs. 100 μA, respectively). Higher currents can lead to vasodilation from other endothelium-dependent factors, such as bradykinin and substance P [35]. Importantly, artefactual vasodilation from high currents is amplified when water is used as the drug vehicle [44], as in the Foster and colleagues study, but is eliminated when 0.5% sodium chloride is used [44], as in the present study. Thus, differences in protocol make comparing the findings of both studies difficult.

In the present study, differences in NO stimulation between the assessments could have contributed to the independence of the microvessels and macrovessels through differential effects on endothelial cell receptors. Whereas SNP, FMD and GTN predominantly evoke maximum NO release [8,43], NO inhibition reduces only 30% to 40% of the microvascular vasodilatory response induced by ACh [45], suggesting that other factors, such as endothelium-derived hyperpolarising factor, may also contribute to vasodilation in the resistance vessels [46]. In addition, application of pharmacologic (ACh and SNP) [47] and physiologic (shear stress) [48] stimuli activate different endothelial receptors [49], and consequently there may be differences in the vasodilatory response between the two assessments.

In the current work, participants were not asked to withhold their antirheumatic drug treatment or vasoactive medications prior to the vascular assessments, as examining patients while they maintain their normal medication regime may provide a better reflection of the patient's arterial condition in an everyday setting. However, additional analyses were conducted to explore the influence of vasoactive medication on the association between microvascular and macrovascular function. Excluding patients receiving vasocative medication (*n *= 46) did not change the findings (data not reported). Another potential limitation of this study is the sample size. However, the effect size of the correlations in the current data is small [50]. This means that, with a power of 0.80 and α set at 0.05, the required sample size to detect a significant association would be 783 participants [50]. Thus, this suggests that the null findings presented in the current study are due to effect size rather than to sample size.

As stated above, assessments of endothelial function have been reported to be good predictors of long-term cardiovascular events in individuals with CVD [12-16] and in healthy older participants [17]. However, in RA, to our knowledge, no studies have examined whether poor endothelial function relates to long-term adverse CV outcomes. Only one study with a relatively small sample size examined the prognostic value of carotid intima media thickness (CIMT), which is an indicator of vascular morphology rather than function [51]. That study showed that patients who experienced a cardiac event during a five-year follow-up period also had greater CIMT at baseline compared to patients without a cardiac event [51]. Therefore, to understand whether and how vascular function is predictive of cardiovascular events, detailed longitudinal assessments are necessary and should include assessment of both the microvasculature and the macrovasculature.

## Conclusions

In summary, the present study has shown that microvascular and macrovascular endothelial function were not associated with each other in patients with RA, suggesting that these assessments cannot be used interchangeably in this population. Assessments of both vascular beds may provide more meaningful clinical information on vascular risk in RA, but this needs to be confirmed in long-term prospective studies.

## Abbreviations

Ach: acetylcholine; CIMT: carotid intima media thickness; CVD: cardiovascular disease; CV: coefficient of variation; DAS28: disease activity score in 28 joints; ED: endothelial dysfunction; FMD: flow-mediated dilation; GTN: glyceryl trinitrate-mediated dilation; LDI: laser Doppler imaging; NO: nitric oxide; RA: rheumatoid arthritis; VIA: vascular imaging analysis.

## Competing interests

The authors declare that they have no competing interests.

## Authors' contributions

AS participated in the design of the study, recruited patients, performed the vascular assessments, conducted data analysis and drafted the manuscript. DC, GK and JVVZ participated in the design of the study and helped with data analysis and drafting the manuscript. All authors read and approved the final manuscript.
